# Interleaved practice enhances memory and problem-solving ability in undergraduate physics

**DOI:** 10.1038/s41539-021-00110-x

**Published:** 2021-11-12

**Authors:** Joshua Samani, Steven C. Pan

**Affiliations:** 1 Department of Physics and Astronomy, University of California, Los Angeles, CA USA; 2 Department of Psychology, University of California, Los Angeles, CA USA

**Keywords:** Human behaviour, Education

## Abstract

We investigated whether continuously alternating between topics during practice, or interleaved practice, improves memory and the ability to solve problems in undergraduate physics. Over 8 weeks, students in two lecture sections of a university-level introductory physics course completed thrice-weekly homework assignments, each containing problems that were interleaved (i.e., alternating topics) or conventionally arranged (i.e., one topic practiced at a time). On two surprise criterial tests containing novel and more challenging problems, students recalled more relevant information and more frequently produced correct solutions after having engaged in interleaved practice (with observed median improvements of 50% on test 1 and 125% on test 2). Despite benefiting more from interleaved practice, students tended to rate the technique as more difficult and incorrectly believed that they learned less from it. Thus, in a domain that entails considerable amounts of problem-solving, replacing conventionally arranged with interleaved homework can (despite perceptions to the contrary) foster longer lasting and more generalizable learning.

## Introduction

In virtually all learning domains, different topics or skills need to be mastered. Examples include derivatives and integrals in calculus, body systems in physiology, and the forehand, backhand, and serve in tennis. An intuitive approach to achieving mastery in such cases is to focus on learning one topic or skill at a time, which cognitive scientists refer to as *blocking* or *massing* (e.g., given concepts A, B, and C, studying three examples of each concept according to an “A_1_A_2_A_3_B_1_B_2_B_3_C_1_C_2_C_3_” schedule). Blocking is ubiquitous throughout education, including in mathematics, science, and language curricula^1–3^. Its use is consistent with the common assumptions that human beings learn best when topics are introduced in isolation^4^, the learning of concepts is facilitated by exposure to successive examples of the same concept^5^, and that repetition practice fosters the development of expertize^6^ (although there are varying perspectives as to the veracity of these assumptions). In contrast, researchers have recently begun investigating an alternative approach known as interleaved practice (henceforth, *interleaving*). Interleaving involves switching between topics (or skills, concepts, categories, etc.) during learning (e.g., studying concepts A, B, and C using an “A_1_B_1_C_1_A_2_B_2_C_2_A_3_B_3_C_3_” schedule)^7^. Consequently, to-be-learned materials are learned in juxtaposition to one another, rather than one at a time. Interleaving may improve attention^8^, induce memory retrieval processes^9^, prompt mental comparison processes^10^, foster relational processing^3^, and simulate the unpredictability of real-world situations^9^, all of which may be beneficial for learning. However, the benefits of interleaving have not yet been extensively explored in authentic educational contexts^11^, and the technique is not generally well known as an effective learning technique among students or instructors^9^. Hence, interleaving is currently rarely used in pedagogical settings^1–3^.

To date, most research on interleaving involves laboratory studies wherein perceptual categories such as artists’ painting styles^12–14^, biological taxonomic classifications^15–17^, or artificial shapes^18–20^ are learned. In these studies, example images of to-be-learned categories are studied in blocked or interleaved fashion, followed by a classification test wherein new images that were drawn from the previously learned categories are shown. Typically, categories that were interleaved are classified more accurately than categories that were blocked^7,20^. A recent meta-analysis found that the typical benefit of interleaving for perceptual category learning is Hedges’ *g* (effect size) = 0.67, 95% confidence interval (CI) [0.57, 0.77] for artists’ paintings and *g* = 0.31, 95% CI [0.17, 0.54] for artificial shapes^8^. The largest interleaving benefits have usually been observed for groups of categories that are perceptually similar (e.g., evolutionarily-related bird families), which implies that interleaving is more effective when to-be-learned materials are confusable with one another^8,21^. Mechanistically, benefits of interleaving for perceptual category learning have been attributed to the temporal spacing between category exemplars that occurs during such interleaving, which constitutes a form of distributed practice (which over a century of research has established can improve memory^22^), as well as learners’ attention being focused on differences between categories (i.e., the attention bias and discriminative contrast framework, wherein interleaving-induced focused attention may yield improvements in the ability to discriminate between perceptually similar categories)^12,13,23,24^.

Based on the aforementioned research, recent reviews have defined the “interleaving effect” as improved inductive learning*—*that is, the mental process of acquiring conceptual knowledge from the study of exemplars—that stems from interleaving exemplars of visual or other perceptual categories^8,11,25^. A question left largely unanswered, however, is whether the interleaving effect extends beyond inductive learning tasks wherein the only determination of category membership is needed. In particular, it has yet to be fully established (a) whether interleaving enhances memory for to-be-learned facts as opposed to perceptual categories, (b) whether interleaving is effective for tasks that require substantial problem-solving, and (c) whether interleaving is effective in authentic educational settings and across extended time intervals^3,9,21^. These questions pertain to many contexts wherein interleaving could be used. As one example, an instructor might choose to interleave a series of different homework problems that require factual knowledge and the execution of stepwise procedures. Initial efforts to address these questions have involved interleaving in such domains as mathematics^21,26,27^, second language instruction^2,28,29^, and other areas^30^.

Thus far, the emerging literature on such uses of interleaving has yielded promising results and especially in the domain of middle-school mathematics. For example, in a 2014 classroom study, the use of interleaved homework assignments to practice algebra and graphing problems (e.g., solving for *x* in an equation; graphing an equation in the form of *y* = *mx* + *b*) yielded subsequent surprise test performance that was nearly double that relative to a condition using blocked homework assignments^21^. Such benefits occurred even for materials that were not necessarily confusable with one another (as featured in most studies of interleaving and perceptual category learning). Even more impressively, a recent randomized controlled trial of interleaved algebra and graphing homework assignments in 54 classrooms (constituting the largest-ever investigation of interleaving to date) reported improvements of Cohen’s *d* (effect size) = 0.83, 95% CI [0.68, 0.97] on surprise delayed tests^31^. These and other results^27,32,33^ raise the prospect that the interleaving effect encompasses more than inductive learning, with potentially broad implications for theories of learning, skill acquisition, and curriculum design.

To further explore the different types of learning that interleaving may promote, the present study examined the effects of interleaving on factual knowledge and problem-solving ability in a previously unexplored domain, namely undergraduate physics. Physics is one of the most popular academic subjects (in the United States alone, ~350,000 undergraduate students take introductory physics courses and over 280,000 high school students take Advanced Placement Physics exams each year)^34,35^. Physics is required not just for physics majors, but also for aspiring professionals in such fields as engineering, medicine, and other areas. Due to the extensive problem-solving skills that are needed, physics is a difficult subject to master, and owing to that difficulty, physics test scores are often among the lowest of all science subjects^34^ (which can cause students to abandon the pursuit of science, technology, engineering, and math (STEM) careers)^36^. Accordingly, there is a pressing need to develop and investigate learning techniques that can be highly effective in physics courses.

The present study addressed that need by conducting a real-world, reasonably well-controlled test of interleaving in undergraduate physics. This test took the form of a preregistered experiment in two large lecture sections of an introductory-level undergraduate physics course (“Physics for Life Science Majors”) at a major US public university. The experiment spanned the first 8 weeks of the 10-week course, during which conventionally blocked homework assignments (wherein, only one problem type is practiced at a time) were replaced with interleaved assignments (involving switching between problem types). Importantly, rather than constructing or selecting materials specifically for research purposes, only the arrangement of homework problems during the course of normal instruction was manipulated and no other aspects of the course were altered. Hence, this test of interleaving occurred in an otherwise “business-as-usual” learning environment, which should increase confidence in its generalizability to real-world settings.

**Fig. 1:**
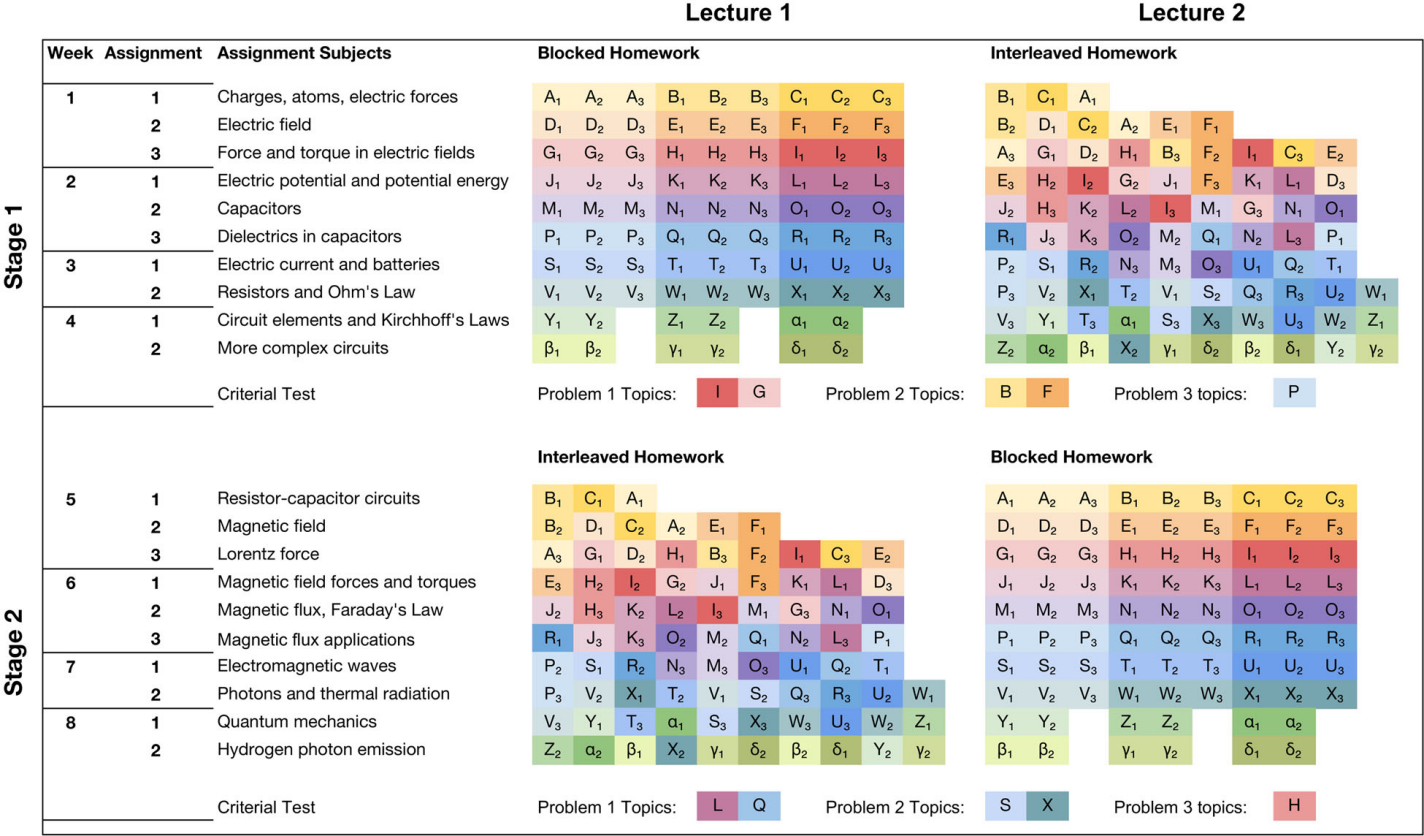
Interleaved versus blocked practice schedules. In each of the two stages of the course, students completed 84 practice problems across 10 homework assignments. Blocked assignments typically featured three successive problems for each of three topics, whereas interleaved assignments typically featured only one problem per topic. In the figure, letters represent topics and subscripts represent the problem number for a given topic (1, 2, or 3). Different topics are also assigned different colors so that it is easier to visually tell them apart. Reflecting the relative simplicity of practicing one topic at a time, topics in each row of the blocked condition correspond perfectly to the assignment subject labeling that row, but this is not the case for the interleaved condition. Topics addressed on the criterial tests are also listed. Due to course time constraints, the last two blocked assignments of each stage include only two problems per topic instead of three. Topics from these assignments were not included in criterial tests.

Across both lecture sections, 350 students participated in a counterbalanced, within-subjects design. During weeks 1–4 (Stage 1), students in the first and second sections (henceforth, Lecture 1 and Lecture 2) received blocked and interleaved homework assignments, respectively, whereas during weeks 5–8 (Stage 2), the assignment types were reversed (see Fig. 1). In other words, Lecture 1 students experienced blocking during Stage 1 and interleaving during Stage 2, whereas Lecture 2 students experienced the reverse. This arrangement ensured that each student in each section ultimately experienced both practice types.

During the course, each of the three weekly lectures was accompanied by a homework assignment. With blocked assignments, each topic was repeatedly practiced in succession with no intervening topics, whereas with interleaved assignments, each successive problem involved a change in the topic (for a list of topics, see Table 1). Of the nine problems per assignment, blocked assignments had three successive isomorphic problems per topic (i.e., having the same underlying problem-solving structure with contrasting surface features), which resembles the arrangement of practice exercises that occurs in many educational contexts^1^, whereas interleaved assignments had only one problem per topic, thus requiring students to engage in switching between topics (with the second and third problems per topic appearing on subsequent assignments). Crucially, within each stage, all students completed the same 84 total problems, with only the arrangement of those problems differing.

**Table 1:** List of topics covered in stages 1 and 2.

Label	Stage 1	Stage 2
A	Atomic structure and Coulomb’s Law	Time constant of discharging capacitor
B	Atomic structure and macroscopic materials	Time to discharge the capacitor by a specified amount
C	Coulomb’s Law and charge conduction	RC Circuits
D	Computing the electric field—like charges	Magnetic field of transmission line—comparisons
E	Computing the electric field—dipole on-axis	Power consumption of MRI solenoid
F	Computing the electric field—charges on different axes	Superposition of straight wire magnetic fields
G	Electric and gravitational force on a point charge	Lorentz force acceleration
H	Torque on a dipole in an electric field	Cyclotron motion in microwave
I	Electric field in a capacitor and charge kinematics	Lorentz force geometric effect on motion
J	Electric potential, potential energy, and work	Magnetic force on a wire balancing gravity
K	Point charge electric potential and energy conservation	Maximum torque of magnetic field on a current loop
M	Electric potential in capacitors	Solenoid magnetic flux through a loop
N	Electric potential of multiple point charges	Magnetic flux through a circular coil
O	Electric potential, field, and force	Faraday’s Law—induced current in a loop
P	Comparing capacitors containing different dielectrics	Magnetic flux through the loop—various geometries
Q	Computing geometric capacitor properties	Faraday’s Law quantitative and qualitative
R	Energy stored and released by capacitors	Ohm’s Law in an MRI machine
S	Current as electron flow	Electric and magnetic fields in a laser
T	Current as positive and negative ion flow	Intensity and magnetic field of radio signals
U	Work done on charges in a battery	Electromagnetic wave penetration depth
V	Comparing resistivities of materials	Photon description of light intensity
W	Computing resistivity of a material	Thermal radiation as photon emission
X	Comparing energy in batteries and in other systems	Comparing power output of thermal light sources
Y	Power consumption in a simple circuit	Computation of de Broglie wavelength
Z	Comparing power consumption in different circuits	Quantum particle in a box
α	Resistance of a composite wire	Photon emission spectrum of a quantum system
β	Power in circuit with parallel and series combined	Hydrogen photon emission spectrum
γ	Circuits with tricky topology—current and power	Hydrogen emission—extreme wavelengths
δ	Circuits with bulbs and resistors—current and power	Hydrogen emission—impossibility questions

Labels correspond to Fig. 1.

To measure the potential effects of interleaving, we administered an in-class surprise criterial test at the conclusion of each stage. These tests followed the approach taken in recent studies of interleaving and mathematics^31,33^ and avoided contaminating effects of cramming, study group activities, and other events that can occur with increasing frequency in the period leading up to pre-announced exams. Both tests featured three novel problems that were more difficult than those included in the homework assignments. The first two problems required integrating concepts and procedures from two separate topics, whereas the third problem required applying a single topic in a new scenario. All three problems required recall and application of factual content conveyed in formulas (see Fig. 1). To derive answers, students had to correctly recognize the topics involved, all of which were last encountered more than 1 week prior; recall relevant formulas, rules, and principles; and in two of three problems, integrate and apply that information to devise a new solution strategy^37^ (which could be viewed as requiring higher-order reasoning, integration, and constructive thought processes as opposed to simply recalling and repeating previously learned information)^38,39^.

As an example, one criterial test problem required recognizing the relevance of both Faraday’s Law and torque on a current loop in a magnetic field, recalling corresponding relevant formulas, and combining them in a novel way to compute the torque on a current loop in the magnetic field of an magnetic resonance imaging machine. Importantly, this combination of problem-solving processes was not included in any of the homework assignments and had not been specifically taught in the course. This type of problem also differed from the isomorphic problems commonly used in prior research on interleaving and problem-solving skills^26,31–33,40^.

## Results

### How did students perform on interleaved versus blocked homework assignments—and how did they perceive both practice types?

Across both lecture sections, 290 students in stage 1 (83% of the total enrolled) and 286 students in Stage 2 (82% of total enrolled) experienced the experimental manipulation in its entirety by completing and turning in all of the homework assignments. Per our preregistered inclusion criteria, only data from those students were analyzed. Although that analysis revealed disparities between interleaving and blocking in terms of student performance, judgments of difficulty, and judgments of pedagogical effectiveness, there was no advance indication of any interleaving benefit.

With respect to overall performance, students correctly solved more blocked than interleaved homework problems (Table 2), with a mean deficit on interleaved assignments of 0.05 and 0.09 proportion correct in Stages 1 and 2, respectively. When interpreting these results, it is important to consider that there were nine different problem types on most interleaved assignments, with each type requiring a different problem-solving strategy, whereas, with most blocked assignments, there were only three problem types. Hence, the expectation that the blocked assignments would be easier was confirmed by student performance.

**Table 2:** Homework assignment accuracy and metacognitive judgment data.

Stage	Measure	Blocked mean (95% CI)	Interleaved mean (95% CI)
1			
	Mean accuracy	0.74 (0.73, 0.75)	0.69 (0.68, 0.70)
	Judgment of difficulty (proportion of “medium” to “difficult” ratings)	0.86 (0.84, 0.87)	0.94 (0.93, 0.95)
	Judgment of learning (proportion of “well” to “extremely well” ratings)	0.57 (0.54, 0.60)	0.51 (0.48, 0.53)
2			
	Mean accuracy	0.76 (0.75, 0.76)	0.67 (0.66, 0.68)
	Judgment of difficulty (proportion of “medium” to “difficult” ratings)	0.81 (0.80, 0.83)	0.89 (0.87, 0.90)
	Judgment of learning (proportion of “well” to “extremely well” ratings)	0.48 (0.45, 0.50)	0.40 (0.39, 0.43)

When asked at the end of each assignment to make metacognitive judgments—that is, to evaluate their own process of learning—students tended to rate interleaved assignments as more challenging and yielding less mastery (Table 2). For both practice types, the largest proportion of students’ judgments of difficulty spanned from the “medium” to “difficult” categories, but a higher proportion of those ratings occurred at the conclusion of interleaved assignments. Correspondingly, for both practice types, the largest proportion of students’ judgments of learning spanned from “well” to “extremely well,” but a higher proportion of those ratings occurred at the conclusion of blocked assignments. Thus, on interleaved assignments, students performed more poorly, experienced greater difficulty, and perceived fewer learning benefits. On the basis of these findings, one might predict that student performance on a delayed test of the practiced topics would suffer.

### How did interleaving and blocking affect learning as measured on the criterial tests?

**Fig. 2:**
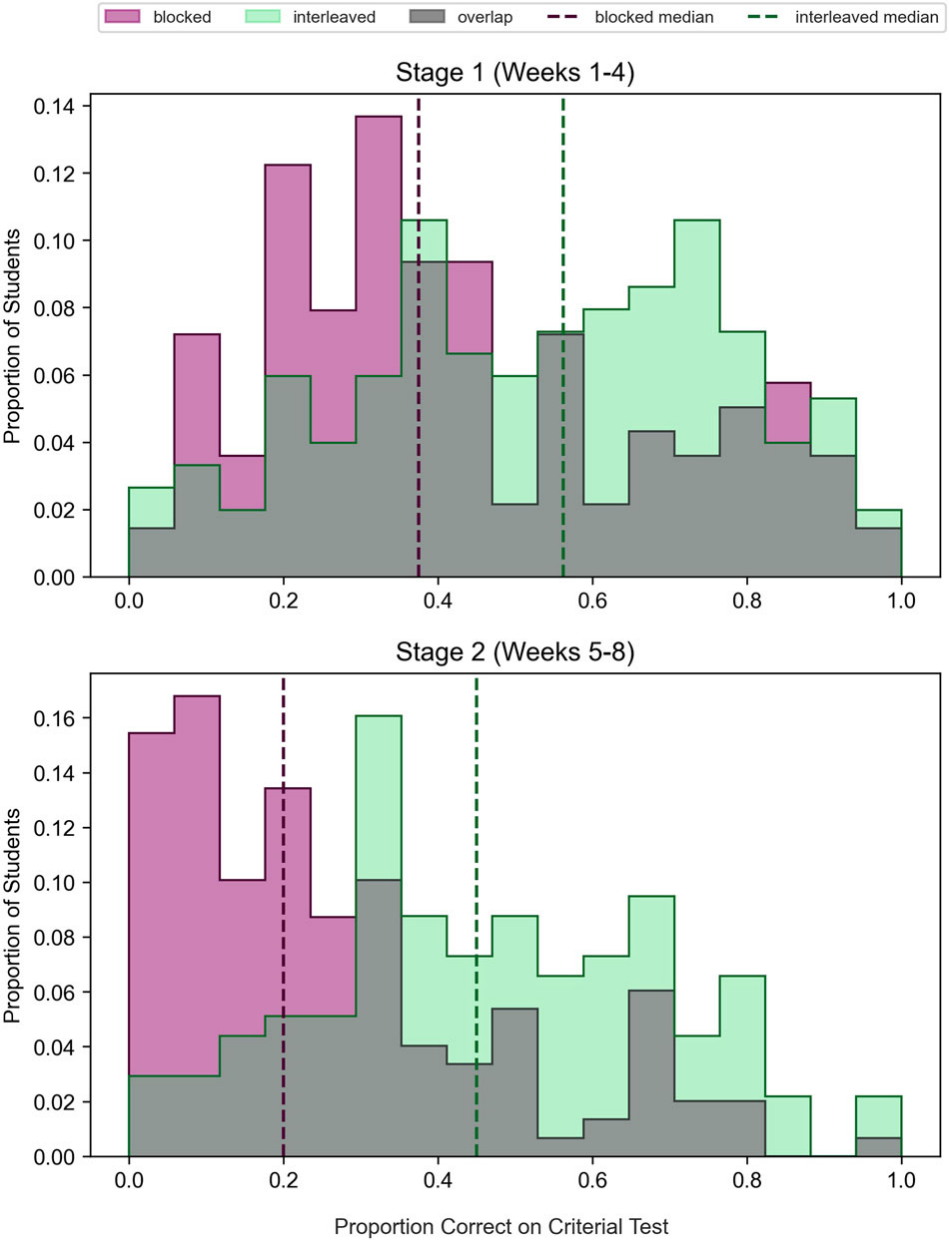
Effect of interleaving versus blocking on criterial test performance. Each histogram displays the distributions of criterial test scores in a given stage, with green representing performance in the interleaved condition and purple representing performance in the blocked condition. The median score in each condition is included as a vertical bar of the corresponding color. Histograms are normalized so that in each condition, the sums of values of all bins equals 1. Mean performance in Stages 1 and 2, respectively, was 0.43 and 0.27 in the blocked condition and 0.54 and 0.47 in the interleaved condition.

Belying the patterns observed on the homework assignments, however, students who had completed interleaved assignments well outperformed those who had completed blocked assignments on the surprise criterial tests. Interleaving yielded higher criterial test scores than blocking in Stage 1, *d* = 0.40, 95% CI [0.17, 0.65], *t*(288) = 3.41, *p* = 0.0008, and in Stage 2, *d* = 0.91, 95% CI [0.66, 1.20], *t*(284) = 7.68, *p* < 0.0001. Thus, interleaving improved the ability to correctly recall and use prior knowledge in an attempt to generate solutions to novel problems. Inspection of the full distributions of test scores further confirms the occurrence of strong interleaving benefits (see Fig. 2). Specifically, interleaving improved median test scores over-blocking by 50% and 125% in Stages 1 and 2, respectively (i.e., interleaving improved learning across both halves of the course and in both counterbalanced groups). In Stage 2, when students had twice as much course content to draw upon (including topics that were arguably more difficult than those that were presented during Stage 1), the effect size of the interleaving advantage was larger.

For additional insights into the effects of interleaving, we examined two distinct sub-measures of test performance: (a) whether students were able to correctly recall necessary formulas, which relies on long-term memory, and (b) whether students’ solution strategies yielded an exact match to the correct answer both in numerical value and in units, which is a more stringent measure of problem-solving ability (as it necessitated devising a multi-step problem-solving strategy and executing its associated computations without making a single error). It should be noted, however, that producing precisely correct answers is uncommon in many introductory-level physics courses due to the inherent conceptual difficulty and computational complexity of the material; in line with that expectation, the mean rate of correct answers, across both conditions, was no >0.34 proportion correct. Sub-measure analyses revealed that interleaving improved long-term memory in Stage 1, *d* = 0.41, 95% CI [0.17, 0.66], *t*(288) = 3.49, *p* = 0.006, and in Stage 2, *d* = 0.96, 95% CI [0.70, 1.24], *t*(284) = 8.05, *p* < 0.0001. Further, interleaving improved the correctness of answers in Stage 1, *d* = 0.25, 95% CI [0.02, 0.48], *t*(288) = 2.17, *p* = 0.0311, and in Stage 2, *d* = 0.40, 95% CI [0.16, 0.64], *t*(284) = 3.32, *p* = 0.0010. Thus, interleaving enhanced both memory and problem-solving accuracy.

Results at the level of individual problems (Table 3) also showed the advantages of interleaving. These advantages were the most consistent (i.e., across both sub-measures) for the easiest problem in each stage (which addressed one as opposed to two topics). Overall, interleaving yielded at least a numerical advantage on both sub-measures for all three problems on both criterial tests.

**Table 3:** Criterial test individual problem results.

Stage	Prob. no.	Topics	Rubric items	Blocked mean (95% CI)	Interleaved mean (95% CI)	Effect size, *p* value
1						
	1	I, G	All	0.44 (0.39, 0.50)	0.51 (0.46, 0.56)	*d* = 0.20*, p* = 0.0974
			Memory only	00.52 (0.47 0.58)	0.59 (0.54, 0.64)	*d* = 0.21*, p* = 0.0755
			Correctness only	0.22 (0.15, 0.29)	0.26 (0.20, 0.34)	*d* = 0.11*, p* = 0.3296
	2	B, F	All	0.45 (0.40, 0.50)	0.51 (0.46, 0.55)	*d* = 0.19*, p* = 0.1104
			Memory only	0.53 (0.47, 0.58)	0.59 (0.54, 0.64)	*d* = 0.19*, p* = 0.1163
			Correctness only	0.07 (0.04, 0.12)	0.10 (0.05, 0.15)	*d* = 0.10*, p* = 0.4053
	3	P	All	0.40 (0.34, 0.47)	0.58 (0.52, 0.64)	*d* = 0.48*, p* < 0.0001
			Memory only	0.45 (0.39, 0.52)	0.64 (0.58, 0.70)	*d* = 0.48*, p* < 0.0001
			Correctness only	0.16 (0.10, 0.22)	0.28 (0.22, 0.36)	*d* = 0.31*, p* = 0.0092
2						
	1	L, Q	All	0.35 (0.31, 0.39)	0.49 (0.45, 0.54)	*d* = 0.60*, p* < 0.0001
			Memory only	0.40 (0.35, 0.44)	0.56 (0.51, 0.60)	*d* = 0.61*, p* < 0.0001
			Correctness only	0.03 (0.01, 0.07)	0.07 (0.03, 0.11)	*d* = 0.15*, p* = 0.2157
	2	S, X	All	0.26 (0.21, 0.30)	0.60 (0.56, 0.65)	*d* = 10.23*, p* < 0.0001
			Memory only	0.30 (0.25, 0.35)	0.70 (0.65, 0.75)	*d* = 10.28*, p* < 0.0001
			Correctness only	0.05 (0.02, 0.09)	0.13 (0.08, 0.19)	*d* = 0.27*, p* = 0.0248
	3	H	All	0.17 (0.12, 0.22)	0.30 (0.24, 0.36)	*d* = 0.40*, p* = 0.0009
			Memory only	0.17 (0.12, 0.21)	0.29 (0.23, 0.34)	*d* = 0.38*, p* = 0.0016
			Correctness only	0.18 (0.12, 0.25)	0.34 (0.26, 0.42)	*d* = 00.38*, p* = 0.0019

Topic labels correspond to Table 1.

### How did interleaving and blocking affect learning and study behaviors in the remainder of the course?

On high-stakes midterm exams occurring 3 days after each criterial test, scores did not significantly differ between the blocked and interleaved conditions (post-Stage 1 midterm, *d* = 0.20, 95% CI [−0.04, 0.43], *t*(288) = 1.68, *p* = 0.0944), and post-Stage 2 midterm, *d* = 0.02, 95% CI [−0.21, 0.25], *t*(284) = 0.16, *p* = 0.8758). Only in Stage 1 was there a hint of an interleaving benefit on the high-stakes exams (as most students did not complete the final exam due to a pandemic-induced campus closure, that exam was not analyzed). Although these patterns suggest a possible limitation on the efficacy of interleaving, there were factors that called into question the diagnosticity of the midterm exams, and these factors led us to include surprise criterial tests as our primary outcome measures. Specifically, exit surveys confirmed that most students engaged in extensive cramming prior to the midterms, but not before the criterial tests (Table 4). Further, the criterial tests were a potentially powerful learning event that previewed the problem format and scope on the midterms and likely influenced students’ study behaviors. These observations are consistent with the fact that the mean proportion correct on midterms (0.74) was high compared with the criterial tests (0.42). Thus, although the benefits of interleaving were not detected on midterm exams, any such benefits may have been occluded by cramming and practice testing.

**Table 4:** Exit survey data.

Question	Choice	Stage 1		Stage 2	
		Blocked	Interleaved	Blocked	Interleaved
Level of surprise (“How surprising was the in-class practice exam?”)					
	Utterly shocking	29.5%	26.5%	16.8%	7.3%
	Surprising	33.1%	41.1%	20.8%	19.7%
	Somewhat surprising	20.9%	17.2%	23.5%	19.0%
	Neither surprising nor unsurprising	5.0%	9.3%	15.4%	21.9%
	Not surprising at all	1.4%	0.7%	18.8%	21.9%
	No response	10.1%	5.3%	4.7%	10.2%
Hours of studying per week before criterial test 1 (2) (“During weeks 1–4 (5–8) until just before the Friday surprise practice test, roughly how many hours on average did you spend each week reviewing for midterm 1 (2)?”)					
	0–3	54.0%	58.3%	49.0%	36.5%
	3–6	25.9%	26.5%	28.2%	30.7%
	6–9	6.5%	7.9%	12.1%	16.8%
	9–12	2.9%	1.2%	4.7%	5.1%
	More than 12	0.7%	0.7%	1.3%	0.7%
	No response	10.1%	5.3%	4.7%	10.2%
Hours of studying between criterial test and the midterm (“During the weekend just after the practice test and just before the midterm, roughly how many hours did you spend studying for the midterm?”)					
	0–5	10.1%	7.3%	6.7%	9.5%
	5–10	18.0%	30.5%	28.2%	20.4%
	10–15	27.3%	29.8%	30.9%	22.6%
	15–20	21.6%	19.2%	17.4%	21.9%
	More than 20	12.9%	7.9%	12.1%	15.3%
	No response	10.1%	5.3%	4.7%	10.2%

With respect to the potential effects of interleaving and blocking on study behaviors, there were no significant self-reported study time differences between the two conditions (Table 3). Rather, the most common pattern across both conditions involved minimal studying prior to the criterial test (≤3 h over 4 weeks) and intense studying between the criterial tests and midterms (≥10 h over 3 days). Such cramming is almost universal among student study behaviors^41^. These patterns suggest that the benefits of interleaving on the criterial tests cannot be attributed to interleaving-induced changes in the volume of studying, but rather to qualitative changes in the learning that occurred during the completion of the homework assignments.

## Discussion

The present results reveal that interleaving can indeed enhance memory and problem-solving ability in the domain of undergraduate physics. Specifically, the use of homework assignments wherein problem types were interleaved, as opposed to conventionally blocked, generated learning improvements on two surprise criterial tests that were comprised of novel and more challenging problems. Such improvements were, in effect size terms, relatively large compared with other pedagogical techniques^42,43^ (despite some variation across stages and across problems) and comparable to interleaving-induced improvements in such domains as middle-school mathematics^31,33^ and second language learning^2,28^. Further, learning benefits were observed (a) for the case of long-term memory for factual content, (b) for the correctness of answers, (c) after retention intervals of at least one to several weeks, and (d) on surprise criterial tests but not on subsequent high-stakes exams. From the perspective of the literature on interleaving and related techniques (e.g., variability during practice)^44–46^, the present results bolster the conclusion that the benefits of alternating between topics or skills during learning extend well beyond the ability to classify perceptual category exemplars; these benefits can also encompass certain problem-solving skills. Moreover, the present results suggest that the avoidance of supposed preconditions for effective learning—including learning topics in isolation^4^, successive exposures to the same concept^5^, and single-session repetition practice^9^—may not be detrimental for learning. Rather, in line with pedagogical perspectives that encourage variability of practice^1,2^, violating those preconditions may in fact enhance learning. That tentative conclusion may validate the practices of instructors that already incorporate some form of interleaving in their homework assignments, but may not necessarily be aware of it as an evidence-supported learning technique.

Several theoretical mechanisms may account for the observed benefits of interleaving. Here, we summarize five candidates. These explanatory accounts are not necessarily mutually exclusive and have been largely drawn from the literature on interleaving, with some adaptations to problem-solving in introductory physics.

First, interleaving may have facilitated inductive learning of problem categories defined by specific physical concepts or principles. These categories, whose correct identification was necessary to solve criterial test problems, are often easily confusable to novice physics learners, who tend to base their problem representations on literal features instead of abstract principles^47^. The course progressed in a hierarchical manner whereby problems across topics commonly shared literal features, but problem classification was never explicitly discussed; hence, any inductive learning of problem categories would most likely have occurred during practice on homework sets. As has been repeatedly demonstrated in the literature (e.g., the attention bias and discriminative contrast framework), inductive learning of confusable perceptual categories is a context wherein interleaving can excel relative to blocking^12,13,23,24^. It is plausible that the interleaved homework sets, which provided more opportunities to compare non-isomorphic problem categories than the blocked homework sets, yielded similar benefits. However, it is important to note that the criterial tests required additional problem-solving steps, including memory retrieval of formulas. As such, inductive learning of problem categories alone might not be sufficient to explain the observed results.

Second, as previously noted, interleaving incorporates distributed practice (i.e., learning spread out over multiple sessions), which is known to improve long-term memory^10^. According to the study-phase retrieval account of the spacing effect, distributed practice during homework sets may have forced students to engage in repeated long-term memory retrieval processes, which are known to enhance the durability and accessibility of memories^3^. In contrast, with blocking, every successive set of three homework problems involved the same topic, thus allowing students to bypass memory retrieval in favor of knowledge temporarily held in working memory (i.e., repeatedly reusing the same solutions). Hence, productive memory retrieval processes may have been attenuated in the blocked condition, potentially reducing the rate of successfully recalling correct formulas on criterial tests, even in the case that the problem solver had achieved a correct conceptual classification of the problem. Other cognitive processes that distributed practice may engage, such as increased encoding of varied contextual cues, may have also had a facilitative effect on learning^48^.

Relatedly, there is evidence in the interleaving literature to support both minimal and major roles of distributed practice depending on the learning context. In the case of perceptual category learning, conditions that feature extensive amounts of distributed practice in the absence of interleaving have failed to yield similar learning benefits^13,15^, which suggests a minimal role, whereas, in studies involving mathematics or second language learning, interleaving schedules that incorporate substantial amounts of distributed practice have yielded larger benefits, which suggests a major role^2,24^. It is important to note, however, that differences in experimental and task design across studies may have also been factors.

A third explanation involves reduced lag-to-test—that is, elapsed time from practice to assessment—in the interleaved versus blocked conditions. In the present study, each interleaved topic was practiced across a 1-week period following its introduction, whereas each blocked topic was practiced only shortly after its introduction. The interleaved condition, therefore, had more recent exposure (by up to 1 week) on at least one topic per problem at the time of the criterial test, although the lags in both conditions were still at least 1–3 weeks long. It should be noted, however, that having students review to-be-tested topics shortly before a criterial test, which might be expected to attenuate differences in lag-to-test, has not eliminated the interleaving benefit in recent math learning studies^33^.

Fourth, by allowing students to mentally compare different types of problems, interleaving may have fostered more relational processing^3^, potentially improving the ability to integrate concepts from superficially distinct problem categories in order to solve criterial test problems that combined non-isomorphic problem types (see Fig. 1). These problem types were merged through shared concepts, such as emitted radiation power, and not recognizing these connections would have rendered the problems unsolvable. Recognition of common concepts may have been more likely in the interleaved condition due to the inclusion of non-isomorphic problem types on each homework assignment, whereas in the blocked condition, students would have had to deliberately juxtapose different homework sets in order to find the relevant connections. The potential for increased relational processing in the interleaved condition might also be described as an instance of material-appropriate processing*—*that is, cognitive processes that match that needed to perform well on a criterial test^49^ (in the present case, integrating non-isomorphic problem types via specific, connecting concepts) and are not redundant with other processes that may already be occurring.

Finally, given that every successive problem on the interleaved homework assignments involved a different topic, interleaving may have given students practice in strategy selection—that is, choosing the correct solution for a given problem from a range of possible options^3,21,50^. In contrast, the predictability of blocked assignments obviated any need to engage in strategy selection (as students could repeatedly use the same solutions with a high degree of success). Proficiency in strategy selection was crucial for all criterial test problems.

It should be reiterated, however, that none of the accounts presented here are mutually exclusive (e.g., improvements in inductive learning of problem categories and/or relational processing may have facilitated better strategy selection), nor was it the purpose of the present study to adjudicate between them. Any or all of these mechanisms may have jointly contributed to the efficacy of interleaving.

Although the present results are quite clear with respect to an interleaving benefit for memory, the results for “far” transfer of learning^37^—which in the present case involved combining information across topics in order to devise new solution strategies—are more equivocal. If such transfer is to be judged based on numerical and unit correctness, then there was, in effect size terms, a smaller benefit of interleaving relative to the recall of relevant formulas and principles. However, a high level of correct responding was not expected, and the correctness sub-measure could not fully capture the degree to which students were able to successfully transfer their learning (i.e., that measure could not account for better, but imperfect, solution strategies). In our view, further research using more fine-grained measures of problem-solving ability (e.g., having students delineate each solution step, which would have required longer test sessions, and then subjecting those steps to analysis) is needed to clarify the potential of interleaving for far transfer and whether the technique is competitive with other transfer-enhancing approaches^47,51^.

The disparity between homework and criterial test data—wherein interleaving initially yielded poorer performance and lower difficulty and efficacy ratings, yet better criterial test performance—illustrates a metacognitive illusion^52^ that may complicate student acceptance of interleaving. That illusion reflects the tendency of human beings to be inaccurate at judging the progress of their own learning and the relative utility of contrasting pedagogical activities (with more effective techniques being judged as less beneficial and vice versa)^53^. In response, instructors might consider additional measures, such as explaining the long-term benefits of interleaving prior to administering homework assignments^54^. Fortunately, there did not seem to be an overtly hostile reception towards interleaving, at least as conveyed to the course instructor, and student evaluations of the course were also relatively unchanged versus prior iterations of the course taught by the same instructor.

From an application standpoint, it is promising that the methods used in the present study were relatively simple and could be adapted to other contexts wherein multiple topics are learned using blocked homework assignments. Simply interleaving those assignments in a similar fashion may greatly enhance their effectiveness. We wish to caution, however, that instructors and researchers will need to be careful in generalizing the present results to cases wherein assignments do not contain multiple isomorphic or nearly isomorphic problems for each topic, and it is unclear whether such interleaving benefits will be apparent on high-stakes exams after extensive cramming (especially when considering the tendency of some laboratory-developed learning interventions to “wash out” in classroom contexts) and practice exams^55^. If no such benefits reliably occur, then that would constitute a notable limitation, particularly if enhancing exam performance was the sole objective. However, it remains to be determined whether a larger interleaving benefit would be observed in cases where practice exams were more substantially different than subsequent high-stakes exams, as well as after high-stakes exams, during which any benefits of cramming may have dissipated. Finally, implementation issues^56^ such as the relative predictability of interleaving schedules^28^ and the point during the learning process that interleaving is introduced^2,21^ remain to be resolved. Given the incipient state of the classroom-focused interleaving literature, real-world uses of interleaving will inevitably involve a certain amount of trial-and-error.

From the perspective of undergraduate physics education and other forms of STEM learning, the present results serve as a proof-of-concept for a relatively low-cost learning intervention (in terms of time required and necessary equipment) that has the potential to yield sizeable learning benefits. The finding that interleaving benefits learning for one of the most challenging subjects that college students have to master, and does so for the case of relatively difficult problem-solving materials, invites a reevaluation of conventional instructional approaches and a greater appreciation for the influence of practice schedules in the development of skills and expertise. Indeed, it is becoming increasingly apparent that there are a variety of educationally authentic contexts in which human learners benefit more from practicing multiple topics from a given domain at one time, rather than practicing one topic at one time.

## Methods

### Preregistration

The study design and analysis plan were preregistered prior to data collection at: https://osf.io/8t4e5/. Of the analyses described in the main text, the preregistered analysis plan contains the only comparison of overall criterial test and midterm exam performance across conditions. All other analyses, including performance on course assignments, accompanying judgments of learning, and exit survey analysis, were planned after preregistration but before data collection and should be regarded as exploratory.

### Participants

Participants were 350 undergraduate students enrolled in either of two back-to-back lecture sections of Physics 5 C (“Physics for Life Sciences Majors: Electricity, Magnetism, and Modern Physics”) at the University of California, Los Angeles (UCLA) in Winter 2020, which began on 6 January 2020 and ended on 20 March 2020. Per the preregistered inclusion criteria, any student that did not complete any homework assignment during Stage 1 (weeks 1–4) or Stage 2 (weeks 5–8) or that did not take the associated criterial test was removed from the data analyses for the corresponding stage of the study. Consequently, in Stage 1, analyses were performed using data from 139 students in the first lecture section and 151 students in the second lecture section (henceforth, referred to as Lecture 1 and Lecture 2, respectively). In Stage 2, 137 students in Lecture 1 and 149 students in Lecture 2 were included in the analyses. Demographic information for all students included in the data analyses is listed in Supplementary Table 1. It should be noted that there was no significant difference in mean GPA between students in Lecture 1 and Lecture 2. Thus, despite the fact that students enrolled in the lecture section of their choice (often based on their schedule of availability and preference for time-of-day), any potential differences in academic aptitude between the students in the two lecture sections were likely to have been negligible.

The study was approved by the UCLA Human Research Protection Program as exempt from formal review. No written informed consent was required for data collected during the course of normal instruction and reported in a fully anonymous and summary fashion as occurs in this manuscript. Informed consent was obtained for any individually identifiable reporting of data, of which there are none in this manuscript.

### Course description

Physics 5 C is a 10-week lower-division course that is the third in a sequence of required physics courses for life sciences majors at UCLA. The official description of the course states that it addresses: “Electrostatics in vacuum and in water. Electricity, circuits, magnetism, quantum, atomic and nuclear physics, radioactivity, with applications to biological and biochemical systems.” In Winter 2020, the course involved thrice-weekly lecture sections of 50 min each (Lecture 1 from 10 to 10:50 AM and Lecture 2 from 11 to 11:50 AM; each student was enrolled in either of those sections), a weekly discussion section with a duration of 50 min, and a weekly laboratory section with a duration of 110 min. Both lecture sections were taught by the first author of this manuscript (J.S.), a faculty member in the Department of Physics and Astronomy at UCLA, on Mondays, Wednesdays, and Fridays. The discussion and laboratory sections, of which there were multiple sections available each week, were taught by graduate teaching assistants and collaborative problem-solving therein was further facilitated by undergraduate learning assistants.

Grading in Physics 5 C during Winter 2020 was determined via participation questions administered during the lecture sections (5%), discussion section assignments (5%), thrice-weekly homework assignments (20%), laboratory activities (15%), and two midterm exams (22.5% each). Participation questions and homework assignments were completed individually, whereas the remaining graded components were completed entirely or partly in groups. A cumulative final exam was originally scheduled and intended to be the most heavily-weighted aspect of the course (30%); however, that exam was removed from the required list of graded components and was made optional due to COVID-19 pandemic-induced suspension of all in-person instruction at UCLA on 11 March 2020. Importantly, the experimental manipulation and all primary measures of interest (i.e., the criterial tests) had been completed and were unaffected by the time in-person instruction was suspended.

### Materials

Study materials are archived at the Open Science Framework (OSF): https://osf.io/8t4e5/. Course materials were drawn from the assigned textbook (University Physics for the Life Sciences by Knight, Jones and Field), which is a common textbook for undergraduate physics courses in the United States. A list of topics covered during weeks 1–8 of the course is presented in Table 1. There were 30 topics per experimental stage. Each lecture covered topics that roughly corresponded to between 1 and 3 sections of the course textbook. Each lecture began with an outline of what was to be learned followed by explanations of key concepts, worked examples, and clicker questions that were often accompanied by peer instruction. Discussion sections consisted of a short review of relevant topics from that week followed by a group exercise involving a single, reasonably challenging corresponding problem on a worksheet. Students were given credit for attending discussion sections and for demonstrating a reasonable level of effort and completion on the weekly problem as judged by their teaching assistant, but discussion worksheets were not scored for correctness. Weekly labs gave students hands-on experience applying course concepts to real physical systems and typically involved materials that had already been covered a week or two beforehand in lecture and on homework assignments.

Both experimental stages featured 10 homework assignments each spread across 4 weeks. There were nine problems per assignment (exceptions included the last two assignments of the blocked condition as well as the first two and last three assignments of the interleaved condition), for a total of 84 homework problems (see Fig. 1 and the main text). There were three isomorphic problems for a given topic (excepting six topics per experimental stage, for which there were two isomorphic problems). It should be noted that given the constraints used to define blocking and interleaving, the interleaved condition had fewer problems on the first two assignments per cycle (given the number of topics introduced to date), and on the final week of a given cycle, the interleaved condition had one additional problem per assignment and up to two problems per topic (but not presented adjacent to one another), with the blocked condition also having fewer than nine problems each. Given the proximity to the end of each cycle and variations in assignment length, topics that appeared on the final week of assignments were not included on the criterial tests.

Each assignment took the form of a multi-page PDF uploaded to Gradescope (a web application for turning in and scoring assignments) on a Monday, Wednesday, or Friday of a given week. Each assignment contained instructions reminding students to complete each assignment on their own, avoid skipping problems, always show their work in the provided spaces (so as to receive completion credit), and clearly indicate their final answers in provided boxes. Each problem type consisted of using a concept and related formulas to compute the values of one or more physical quantities. Isomorphs for each problem type was generated by varying superficial features that left the underlying computational and conceptual structure invariant, such as by changing values for given physical quantities or changing the context in which the given information was presented.

The final page of each assignment contained three multiple-choice survey questions: (a) How difficult did you find the questions on this assignment?; (b) Over how many days did you complete this assignment?; and (c) How well do you think you have learned the concepts and procedures addressed by these problems?

There were three assignments each week except in weeks 3 and 7, during which there was no class on Monday owing to a holiday. This holiday fell on precisely the same day in the practice schedule during each stage, so the two stages had identical problem set schedules despite the holidays.

Both criterial tests were intended to be completed within a 50-min lecture period and contained three questions each. The formatting of the tests, which were administered in pen (or pencil)-and-paper form, mirrored the homework assignments in that there were provided spaces and boxes to show work and to indicate final answers. Critically, however, the criterial test problems required integrating knowledge from two separately-learned topics, or applying knowledge regarding a previously learned topic in a new way (as described in the main text). The topics addressed on the criterial tests are noted in Fig. 1 of the main text. Given the deviations in the number of problems per assignment in the final week of each cycle, as well as the proximity in time between instruction and the criterial test, all topics addressed in that week were not covered on the criterial tests.

Two midterm exams were administered (both occurring on the first Monday after the end of an experimental stage and ~72 h after the criterial test). Each midterm exam contained five problems that were of a similar type as those presented on the criterial tests.

At the end of the course, students were asked to complete an online exit survey in exchange for extra credit. The survey contained questions addressing (a) how the homework assignments were completed; (b) study activities that occurred prior to the surprise and midterm exams; (c) level of surprise in the surprise exams, and (d) prior physics courses. Questions addressing (a–c) were posed separately for Stages 1 and 2. A complete copy of the exit survey is archived at the aforementioned OSF link.

### Design and procedure

A 2 × 2 counterbalanced design was used with within-subjects factors of condition (blocked vs. interleaved) and Stage (1 vs. 2). Blocking versus interleaving was manipulated by having one lecture section experience blocking and interleaving during Stages 1 and 2, respectively, whereas the other section experienced the reverse of that arrangement. The experiment was implemented as part of regular course activities as follows. On the first day of class, the instructor outlined course expectations as described in the syllabus. The substantial contribution of homework assignments to the course grade was emphasized (and to further incentivize completion of homework assignments, an additional 1% extra credit bonus was promised to all students that completed every single homework assignment). Homework assignments were then released regularly online on each Monday, Wednesday, and Friday (during weeks 1–4 and 5–8, and excepting the Friday of weeks 4 and 8). Each assignment was to be completed within 72 h of it being made available, and finished assignments were to be scanned and uploaded to Gradescope for grading. Fully worked solutions and answers for each assignment were posted each Sunday evening. Grades, rubrics, and answer keys for each assignment were posted on Gradescope within roughly 1 week of the due date. All other course activities, including the lectures, discussion sections, and lab sections, proceeded as per standard practice. The course instructor delivered identical lecture content to both sections throughout the entire course.

During the lecture sections on the Fridays of weeks 4 and 8, the surprise criterial test was administered. That lecture had been billed as a “review session” addressing the content covered over the preceding 4 weeks, with students incentivized to attend by a promise of 1% extra credit. In place of a review session, however, the test was handed out, students were told that they would get up to 1% extra credit according to their performance on the test (although during the actual assignment of grades, all students were given the full 1% extra credit), and students were then given the full 50-minute lecture period to complete the test. Aside from increasing every student’s final grade by 1%, the criterial tests did not impact student grades. The test was proctored by the course instructor and teaching assistants. Survey data revealed that the majority of students were surprised that the “review session” actually entailed a criterial test (see Table 4).

### Measures

Performance on the blocked and interleaved homework assignments was analyzed to provide insights into the relative difficulty of the two learning schedules used. To facilitate analysis, each students’ intended answers, as indicated by entry into provided answer boxes, were transcribed into an electronic spreadsheet, and the answers were then computer-scored against a correct answer list. In all cases, the transcription of homework data was conducted by research assistants that were blind to the condition. In addition, the answers to the three multiple-choice survey questions on each assignment were also transcribed by hand.

Performance on the criterial tests was the primary outcome of interest given that the criterial tests were the purest measures of the effects of the experimental manipulation (i.e., uncontaminated by any additional study or review activities, or foreknowledge of the question types). Every problem on the criterial tests required (a) recognizing which mathematical relationships (often equations) were relevant for solving that problem, (b) writing those relationships down, and (c) appropriately combining them with given values of physical quantities to compute a single final numerical answer with a corresponding physical unit. A rubric based on that employed throughout lower-division physics courses at UCLA was used to score the criterial tests and allowed for inferring whether steps (a–c) were successfully completed. The rubric items per problem fell into two mutually exclusive, exhaustive categories: In the first, “memory” category, each item indicated whether or not one of the necessary equations was recalled and written down correctly; in the second, “correctness” category, each item indicated whether or not the final numerical answer and unit were correct. Criterial tests were each scored by at least two trained raters that were blind to the condition. Each rubric item for each problem was first scored independently by two scorers, after which a third scorer independently scored only those items on which the original two scorers differed. For each rubric item, inter-rater reliability (IRR) between the original two raters was assessed. In Stages 1 and 2, the mean IRR across all rubric items on the criterial test was Cohen’s *κ* = 0.81 and 0.83, respectively.

Null hypothesis significance testing of criterial test data was conducted using *t* tests as per our preregistered analysis plan. All tests were two-tailed. Effect sizes were reported in terms of Cohen’s *d* as defined in prior work^57^. As a supplement to the *t* tests, permutation tests (which do not require the assumption of normality of underlying population distributions) were also conducted. The permutation tests yielded negligibly different *p* values relative to the *t* tests and are not detailed further for simplicity.

Performance on the midterm and final exams were originally to be analyzed separately. Performance on these exams would have reflected the effects of the experimental manipulation as well as review and study activities, including cramming, prior to the exams. However, as the final exam was made optional (and switched to take-home format) due to the COVID-19 pandemic, data for that exam were not available for the vast majority of students. Hence, the analysis of that exam was dropped. Per procedures that the instructor had used in prior physics courses, the midterm exams—which were completed at separate exam periods outside of normal lecture hours—were completed in individual and group stages (i.e., students first attempted the questions on their own, they were organized into groups to share ideas and revise their answers). The results reported in the main text reflect data from the individual stages. The midterm exams were scored by teaching assistants that were also blind to condition.

The exit surveys, which provided additional context for interpreting the study results, were transcribed by research assistants that were blind to condition.

### Reporting summary

Further information on research design is available in the Nature Research Reporting Summary linked to this article.

## Supplementary information

Supplementary Information

Reporting Summary

## Data Availability

Data and materials are archived at the OSF: https://osf.io/8t4e5/.

## Code availability

Analysis code is available upon request.
