# Supplementary material for: Interleaved practice enhances memory and problem-solving ability in undergraduate physics
Source: NPJ Sci Learn. 2021 Nov 12;6:32. doi: 10.1038/s41539-021-00110-x (PMC8589969; doi:10.1038/s41539-021-00110-x)
Supplement: Supplementary file 1 — Supplementary Information [file 41539_2021_110_MOESM1_ESM.pdf]

Supplementary Table 1. Demographic Information for Participants in Stages 1 and 2 separated by condition

Category	Characteristic	Stage 1		Stage 2	
		Blocked	Interleaved	Blocked	Interleaved
**Sample size**					
	Total	139	151	149	137
**Gender**					
	Female	77.0%	57.6%	56.4%	77.4%
	Male	21.6%	41.7%	41.6%	21.9%
	Unknown	1.4%	0.7%	2.0%	0.7%
**Classification**					
	Senior	82.0%	71.5%	72.5%	81.0%
	Junior	18.0%	27.8%	26.8%	19.0%
	Sophomore	0.0%	0.7%	0.7%	0.0%
	Freshman	0.0%	0.0%	0.0%	0.0%
**Grade point average**					
	Mean	3.56	3.57	3.58	3.51
**Admit Status**					
	Freshman admit	89.2%	87.4%	86.6%	89.8%
	Transfer admit	10.1%	11.9%	11.4%	9.5%
	Unknown	0.7%	0.7%	2.0%	0.7%
**Race and International Status**					
	Asian or Pacific Islander	35.3%	39.1%	38.3%	35.0%
	White Non-Hispanic	27.3%	26.5%	26.8%	27.0%
	Hispanic or Latinx	20.1%	21.1%	20.1%	21.9%
	Unstated, Unknown, Other	4.3%	6.0%	6.7%	3.6%
	Foreign	5.8%	4.6%	4.0%	5.8%
	Black Non-Hispanic	5.0%	2.0%	2.0%	4.4%
	American Indian or Alaskan Native	1.4%	0.0%	0.0%	1.5%
	Unknown	0.7%	0.7%	2.0%	0.7%
**Academic major**					
	Biology	18.0%	22.5%	23.5%	20.4%
	Pre-psychobiology	20.1%	16.6%	14.8%	18.2%
	Molecular, Cell, and Developmental Biology	11.5%	9.3%	8.1%	10.2%
	Neuroscience	5.0%	10.6%	12.1%	2.8%
	Environmental Science	9.4%	6.0%	5.4%	10.2%
	Physiological Science	7.2%	7.9%	8.1%	7.3%
	Biochemistry	9.4%	3.3%	4.0%	9.5%
	Microbiology, Immunology, and Molecular Genetics	3.6%	6.6%	8.1%	2.9%
	Human Biology and Society – BS	2.2%	4.0%	3.4%	2.2%
	Pre-computational and Systems Biology	4.3%	1.3%	1.3%	4.4%
	Prehuman Biology and Society – BS	1.4%	2.0%	2.0%	1.5%
	Marine Biology	2.9%	0.0%	0.0%	1.5%
	Ecology, Behavior, and Evolution	0.0%	2.0%	2.0%	0.0%
	Anthropology – BS	0.0%	2.0%	2.0%	0.0%
	Chemistry	1.4%	0.0%	0.0%	1.5%
	History	0.0%	1.3%	1.3%	0.0%

Undeclared – Life Science	0.0%	1.3%	1.3%	0.0%
Pre-Cognitive Science	0.0%	1.3%	1.3%	0.0%
Undeclared	0.7%	0.0%	0.0%	0.7%
Classical Civilization	0.7%	0.0%	0.0%	0.7%
Psychology	0.7%	0.0%	0.0%	0.7%
Pre-Psychology	0.7%	0.0%	0.0%	0.7%
Biophysics	0.0%	0.7%	0.7%	0.0%
Pre-Mathematics for Teaching	0.7%	0.0%	0.0%	0.7%
Mathematics for Teaching	0.0%	0.7%	0.0%	0.0%
Undeclared – Physical Science	0.0%	0.7%	0.7%	0.0%
Chemistry/Materials Science	0.0%	0.0%	0.0%	0.7%
