# Supplementary material for: Interleaved practice enhances memory and problem-solving ability in undergraduate physics
Source: NPJ Sci Learn. 2021 Nov 12;6:32. doi: 10.1038/s41539-021-00110-x (PMC8589969; doi:10.1038/s41539-021-00110-x)
Supplement: Supplementary file 2 — Reporting Summary [file 41539_2021_110_MOESM2_ESM.pdf]

nature research

Corresponding author(s): Joshua Samani, PhD; Steven C. Pan, PhD

Last updated by author(s): Oct 9, 2021

Reporting Summary

Nature Research wishes to improve the reproducibility of the work that we publish. This form provides structure for consistency and transparency in reporting. For further information on Nature Research policies, see our Editorial Policies and the Editorial Policy Checklist.

Statistics

For all statistical analyses, confirm that the following items are present in the figure legend, table legend, main text, or Methods section.

n/a	Confirmed	
☐	☒	The exact sample size (n) for each experimental group/condition, given as a discrete number and unit of measurement
☐	☒	A statement on whether measurements were taken from distinct samples or whether the same sample was measured repeatedly
☐	☒	The statistical test(s) used AND whether they are one- or two-sided<br>*Only common tests should be described solely by name; describe more complex techniques in the Methods section.*
☒	☐	A description of all covariates tested
☐	☒	A description of any assumptions or corrections, such as tests of normality and adjustment for multiple comparisons
☐	☒	A full description of the statistical parameters including central tendency (e.g. means) or other basic estimates (e.g. regression coefficient) AND variation (e.g. standard deviation) or associated estimates of uncertainty (e.g. confidence intervals)
☐	☒	For null hypothesis testing, the test statistic (e.g. F, t, r) with confidence intervals, effect sizes, degrees of freedom and P value noted<br>*Give P values as exact values whenever suitable.*
☒	☐	For Bayesian analysis, information on the choice of priors and Markov chain Monte Carlo settings
☒	☐	For hierarchical and complex designs, identification of the appropriate level for tests and full reporting of outcomes
☐	☒	Estimates of effect sizes (e.g. Cohen's d, Pearson's r), indicating how they were calculated

Our web collection on statistics for biologists contains articles on many of the points above.

Software and code

Policy information about availability of computer code

Data collection	No software was used for data collection. All data was collected using pen (or pencil) and paper materials.
Data analysis	All analyses were performed using the Python 3 programming language and associated open-source libraries.

For manuscripts utilizing custom algorithms or software that are central to the research but not yet described in published literature, software must be made available to editors and reviewers. We strongly encourage code deposition in a community repository (e.g. GitHub). See the Nature Research guidelines for submitting code & software for further information.

Data

Policy information about availability of data

All manuscripts must include a data availability statement. This statement should provide the following information, where applicable:

- Accession codes, unique identifiers, or web links for publicly available datasets
- A list of figures that have associated raw data
- A description of any restrictions on data availability

Anonymized data and materials are archived at the Open Science Framework (OSF): https://osf.io/8t4e5/?view_only=8c8a1a30b2b246cea3fb4ea187acf3b9.

Field-specific reporting

Please select the one below that is the best fit for your research. If you are not sure, read the appropriate sections before making your selection.

☐ Life sciences ☒ Behavioural & social sciences ☐ Ecological, evolutionary & environmental sciences

For a reference copy of the document with all sections, see nature.com/documents/nr-reporting-summary-flat.pdf

Behavioural & social sciences study design

All studies must disclose on these points even when the disclosure is negative.

Study description	Quantitative experimental
Research sample	University of California, Los Angeles undergraduates
Sampling strategy	Sample size was determined by the enrollment limit of the lecture sections in which the experiment took place. The enrollment limit of Physics 5C at UCLA is 180 per lecture section. As the experiment was conducted in two lecture sections of Physics 5C, we expected a maximum sample size of 360. Such sample sizes are considered within the range of acceptability for classroom intervention research.
Data collection	Data was collected using pen (and paper) materials. The researcher was present for the data collection periods involving the criterial and midterm tests, but not the homework assignments. The researcher was not blind during the data collection periods, but data scoring was conducted on blinded materials.
Timing	Start date: January 6, 2020. End date: March 11, 2020.
Data exclusions	Our preregistered exclusion criteria were: any student that did not complete any homework assignment during Stage 1 (weeks 1-4) or Stage 2 (weeks 5-8) or that did not take the associated criterial test was removed from the data analyses for the corresponding stage of the study. Consequently, 60 students and 64 students were removed from analysis for Stages 1 and 2, respectively.
Non-participation	No participants dropped out or declined participation.
Randomization	We employed a within-subjects, counterbalanced design wherein all participants experienced both levels of the independent variable (interleaving versus blocking). Participants were assigned at the level of lecture section, with each section receiving one level of the independent variable in the first four weeks, and the other level in the second four weeks.

Reporting for specific materials, systems and methods

We require information from authors about some types of materials, experimental systems and methods used in many studies. Here, indicate whether each material, system or method listed is relevant to your study. If you are not sure if a list item applies to your research, read the appropriate section before selecting a response.

Materials & experimental systems

n/a	Involved in the study	
☒	☐	Antibodies
☒	☐	Eukaryotic cell lines
☒	☐	Palaeontology and archaeology
☒	☐	Animals and other organisms
☐	☒	Human research participants
☒	☐	Clinical data
☒	☐	Dual use research of concern

Methods

n/a	Involved in the study	
☒	☐	ChIP-seq
☒	☐	Flow cytometry
☒	☐	MRI-based neuroimaging

Human research participants

Policy information about studies involving human research participants

Population characteristics	See above; no covariates were used in any of the data analyses.
Recruitment	Participants were all enrolled students in Physics 5C during Winter 2020.
Ethics oversight	UCLA Human Research Protection Program

Note that full information on the approval of the study protocol must also be provided in the manuscript.
